# Finding Your Mate at a Cocktail Party: Frequency Separation Promotes Auditory Stream Segregation of Concurrent Voices in Multi-Species Frog Choruses

**DOI:** 10.1371/journal.pone.0021191

**Published:** 2011-06-15

**Authors:** Vivek Nityananda, Mark A. Bee

**Affiliations:** Department of Ecology, Evolution, and Behavior, University of Minnesota, Twin Cities, St. Paul, Minnesota, United States of America; Claremont Colleges, United States of America

## Abstract

Vocal communication in crowded social environments is a difficult problem for both humans and nonhuman animals. Yet many important social behaviors require listeners to detect, recognize, and discriminate among signals in a complex acoustic milieu comprising the overlapping signals of multiple individuals, often of multiple species. Humans exploit a relatively small number of acoustic cues to segregate overlapping voices (as well as other mixtures of concurrent sounds, like polyphonic music). By comparison, we know little about how nonhuman animals are adapted to solve similar communication problems. One important cue enabling source segregation in human speech communication is that of frequency separation between concurrent voices: differences in frequency promote perceptual segregation of overlapping voices into separate “auditory streams” that can be followed through time. In this study, we show that frequency separation (ΔF) also enables frogs to segregate concurrent vocalizations, such as those routinely encountered in mixed-species breeding choruses. We presented female gray treefrogs (*Hyla chrysoscelis*) with a pulsed target signal (simulating an attractive conspecific call) in the presence of a continuous stream of distractor pulses (simulating an overlapping, unattractive heterospecific call). When the ΔF between target and distractor was small (e.g., ≤3 semitones), females exhibited low levels of responsiveness, indicating a failure to recognize the target as an attractive signal when the distractor had a similar frequency. Subjects became increasingly more responsive to the target, as indicated by shorter latencies for phonotaxis, as the ΔF between target and distractor increased (e.g., ΔF = 6–12 semitones). These results support the conclusion that gray treefrogs, like humans, can exploit frequency separation as a perceptual cue to segregate concurrent voices in noisy social environments. The ability of these frogs to segregate concurrent voices based on frequency separation may involve ancient hearing mechanisms for source segregation shared with humans and other vertebrates.

## Introduction

Hearing requires the analysis of acoustic scenes comprising multiple, concurrent sounds and the assignment of different sounds to their correct sources [1], [2], [3]. This is a non-trivial problem for the auditory system because each ear receives a composite pressure wave representing the often-complex mixtures of sounds in the environment. The auditory system must parse this raw sensory input to construct perceptual representations of individual sound sources, a process often referred to as “auditory scene analysis” [4]. A particularly well-studied problem of sound source segregation in humans involves our ability to perceive speech in noisy social gatherings with multiple talkers and competing voices. Understanding how auditory systems solve this so-called “cocktail party problem” [5], [6] has important implications for key issues in human health and technology, such as the development of improved hearing aids, cochlear implants, and speech recognition software [6]. The human auditory system appears to exploit a relatively small number of perceptual cues in the analysis of acoustic scenes [1], [2], [3], [4], [6], [7]. Our ability to segregate temporally overlapping voices into separate “auditory streams” based on a difference in their fundamental frequencies, or perceived pitch, is well established [8], [9], [10]. Likewise, psychophysical studies using simple melodies or sequences of two interleaved tones differing in frequency (e.g., ABABAB…) confirm the robust abilities of spectral separation to promote the segregation of temporally overlapping or interleaved sounds into separate auditory streams in humans (e.g., A–A–A–… and –B–B–B…) (reviewed in [11]). But what about other vocally communicating animals?

The cocktail party problem is not unique to humans and our machines. Nonhuman animals in a diversity of taxa have social systems in which they encounter – and solve – evolutionarily analogous communication problems. This is especially true of species that rely on acoustic signaling in dense aggregations, such as colonies and choruses [12], [13]. But we know very little about how nonhuman animals segregate overlapping voices in these sorts of social environments [12], [13]. In this study, we investigated how frogs solve a cocktail-party-like communication problem. Frogs are well known for forming dense breeding choruses in which males produce loud, advertisement calls to attract females (reviews in [14], [15]). Choruses often comprise hundreds of simultaneously calling individuals of multiple different species, each with a unique vocal repertoire. Successful reproduction requires that females detect, recognize, and localize the vocalizations of a conspecific male amid the cacophony generated by these mixed-species choruses [14]. However, the noise generated in a chorus and interference from overlapping calls (both heterospecific and conspecific) can constrain a female's perception of vocalizations and lead to evolutionarily costly errors and non-optimal choices of mates [16], [17], [18], [19]. Thus female frogs must often overcome a *multi-species* cocktail-party-like problem to reproduce successfully. Investigations into how frogs perceive acoustic signals in noisy social environments are particularly important from a comparative perspective because of the uniqueness of their auditory systems [reviewed in 14,15]: frog ears function as pressure-difference receivers, the amphibian inner ear is unique among vertebrates in having two anatomically distinct sensory papillae that encode different frequency ranges of airborne sounds, and frogs lack auditory cortex.

**Figure 1:**
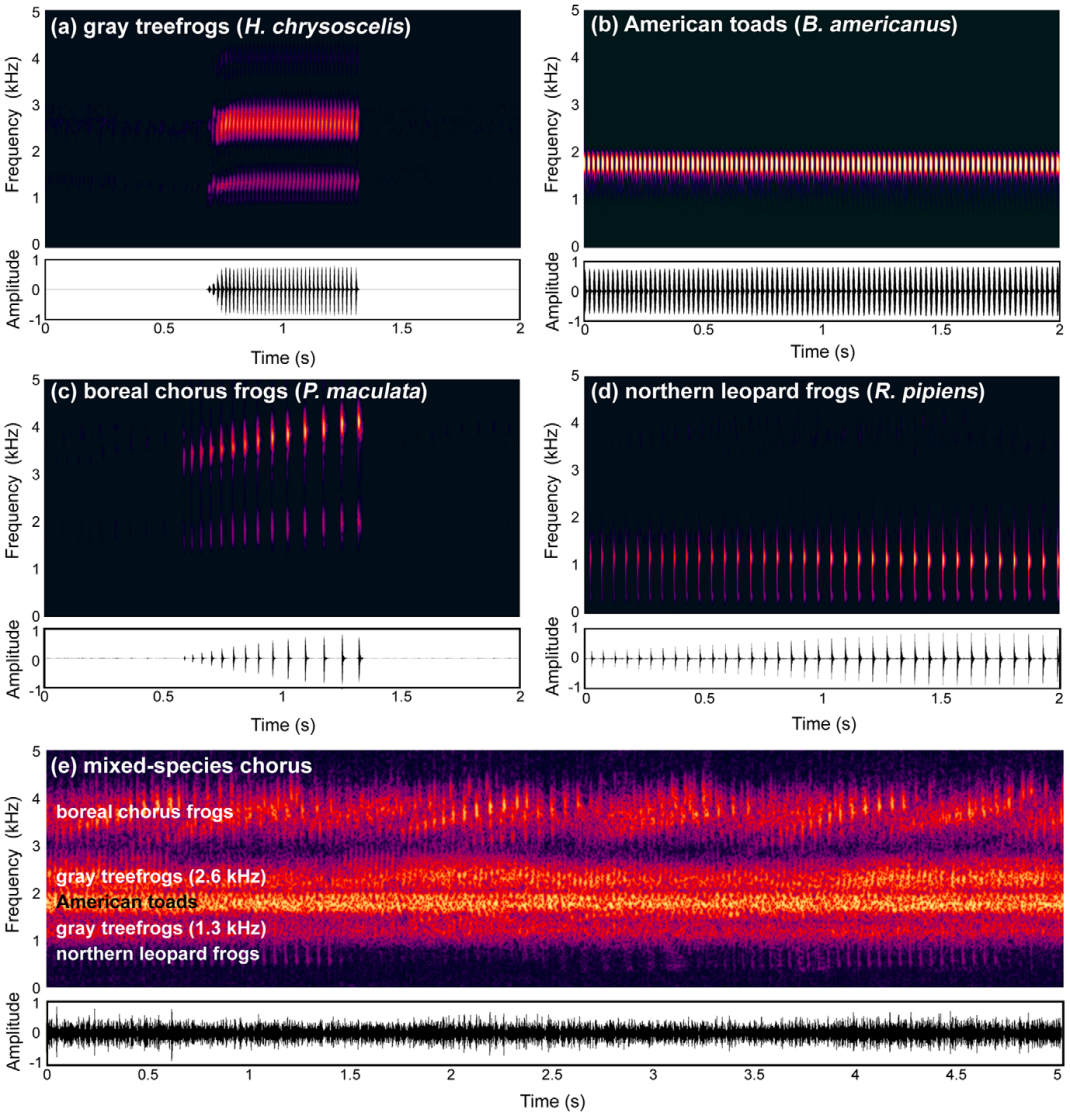
The acoustic scene of a mixed-species breeding chorus. Spectrograms (top traces) show frequency as a function of time (amplitude shown as color intensity) and oscillograms (bottom traces) show amplitude as a function of time. In Minnesota, U.S.A., where our study was conducted, three heterospecific species that form mixed-species choruses with gray treefrogs are boreal chorus frogs, American toads, and northern leopard frogs. (*a*) The advertisement call of a male gray treefrog (*Hyla chrysoscelis*) (see text for description). (*b*) American toads (*Bufo americanus*) produce a long (≈5–50 s), trilled call (≈35–45 pulses s^−1^) with a single spectral component (≈1.7–2.0 kHz) that falls between the two spectral components of the gray treefrog call [53]; a 2-s segment of a longer call is shown here. (*c*) Boreal chorus frogs (*Pseudacris maculata*) produce a pulsed advertisement call of approximately 750–950 ms in duration (≈13–18 pulses s^−1^) and with a bimodal frequency spectrum having peaks at about 1.9 kHz (−8 to −22 dB) and 3.8 kHz (0 dB) [54]. (*d*) Northern leopard frogs (*Rana pipiens*) also produce a relatively long (≈2–5 s), trilled call (termed a “snore”) that is fairly broadband (≈0.5–2.0 kHz), with dominant frequencies ranging from about 0.9 to 1.5 kHz [55]. (*e*) A mixed-species chorus in Minnesota comprising calls by all four species depicted in (*a*–*d*). All recordings were made with Sennheiser microphones (ME66 or ME67) and a Marantz PMD670 recorder. Spectrograms were generated using an FFT window size of 1024 points with Blackman-Harris windows.

Quite commonly, the syntopically and synchronously breeding frogs composing mixed-species choruses have calls with different frequency spectra [20], [21]. Here, we tested the hypothesis that females of Cope's gray treefrog (*Hyla chrysoscelis*) exploit these frequency differences between the competing voices of different frog species as a cue for perceptually segregating concurrent sources in mixed-species choruses. Male gray treefrogs produce a short call (≈600–800 ms) composed of discrete pulses (≈24–40 pulses/call) produced at rates of about 40–50 pulses s^−1^ (Fig. 1a). Each pulse has a “bimodal” frequency spectrum with acoustic energy contained in two spectral components with frequencies (and relative amplitudes) of about 1.3 kHz (−6 to −10 dB) and 2.6 kHz (0 dB). Each spectral component is primarily encoded by a different inner ear papilla (the amphibian and basilar papillae, respectively) [22]. Across their geographic range, gray treefrogs breed synchronously and syntopically with numerous other frog species (Fig. 1b–d; [23]). In many instances, the males of heterospecific frog species produce concurrent, pulsatile vocalizations (see Fig. 1e). Our study addressed two questions: Do the calls of heterospecific species contribute to the gray treefrog's cocktail-party-like problem? If so, could gray treefrogs exploit frequency separation between overlapping voices to segregate the calls of conspecific males from those of other syntopically and synchronously breeding species?

## Results

### Can Gray Treefrogs Hear the Acoustic Frequencies in Heterospecific Calls?

**Figure 2:**
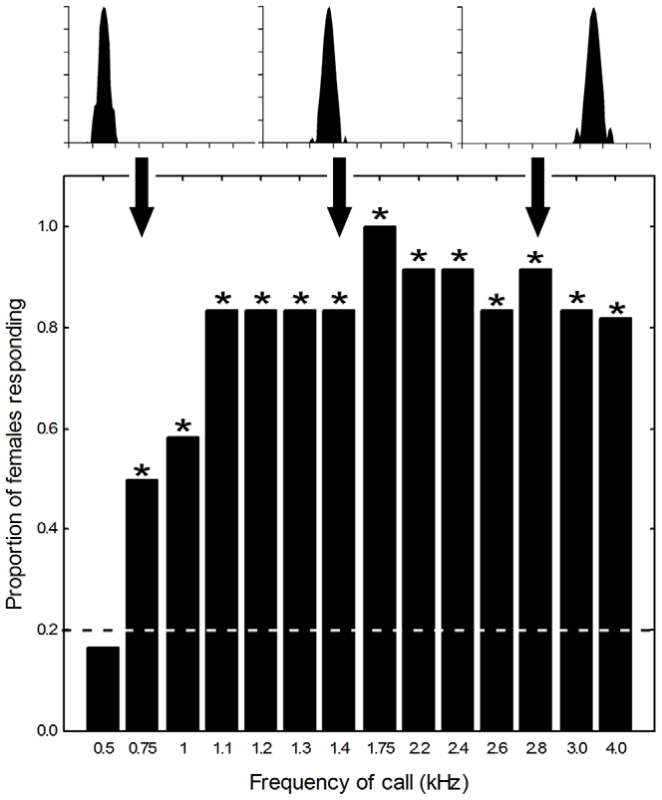
Results from no-choice tests of audibility. Depicted are the proportions of subjects that responded to unimodal calls presented at 67 dB SPL with carrier frequencies as indicated along the x-axis. Insets depict the power spectra of three selected stimuli showing relative amplitude (from 0 dB to −36 dB in 6-dB steps; y axis) as a function of frequency (from 0 to 4 kHz, 0.5-kHz steps; x-axis). The sample size for each bar was n = 12 for all stimuli except that at 4.0 kHz, for which the sample size was n = 11. Asterisks indicate significant differences (p<0.05) in one-tailed binomial tests of the hypothesis that the represented proportion exceeded the expected null proportion of 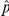 = 0.2 (dashed line).

We conducted an initial audibility experiment to determine whether, as predicted from midbrain audiograms [24], gray treefrogs hear the frequencies emphasized in the calls of heterospecifics breeding in mixed-species choruses (Fig. 1). If so, the calls of heterospecific frog species would be expected to contribute to the magnitude of the gray treefrog's cocktail-party-like problem. The significance of this question as the starting point for our study stems from the traditional notion that the frog's peripheral auditory system functions as a “matched filter” [14], [25] that is tuned to the frequencies present in conspecific calls in order to filter out heterospecific calls with different frequencies. Using no-choice phonotaxis tests [26], we presented females with a synthetic target signal with the average gross-temporal properties of conspecific calls. Previous studies have shown calls with these temporal properties to be effective at eliciting positive phonotaxis [26], [27], [28]. The signal was presented at 67 dB SPL and had a “unimodal” frequency spectrum comprising a single carrier frequency that varied across separate tests between 0.5 kHz and 4.0 kHz. Our prediction was that signals with audible carrier frequencies would elicit positive phonotaxis. Readers should note that this was a conservative test of audibility, because it was possible that signals could be audible but unattractive and thus fail to elicit phonotaxis. As illustrated in Fig. 2, females approached signals with carrier frequencies between 0.75 kHz and 4.0 kHz significantly more often than expected by chance. This result confirmed that frequencies emphasized in the calls of other frog species in mixed-species choruses are audible (cf. Figs. 1 and 2); hence, the temporally overlapping voices of heterospecific species are sound sources that potentially contribute to the gray treefrog's cocktail-party-like problem.

### Does Frequency Separation (ΔF) Promote Perceptual Segregation?

**Figure 3:**
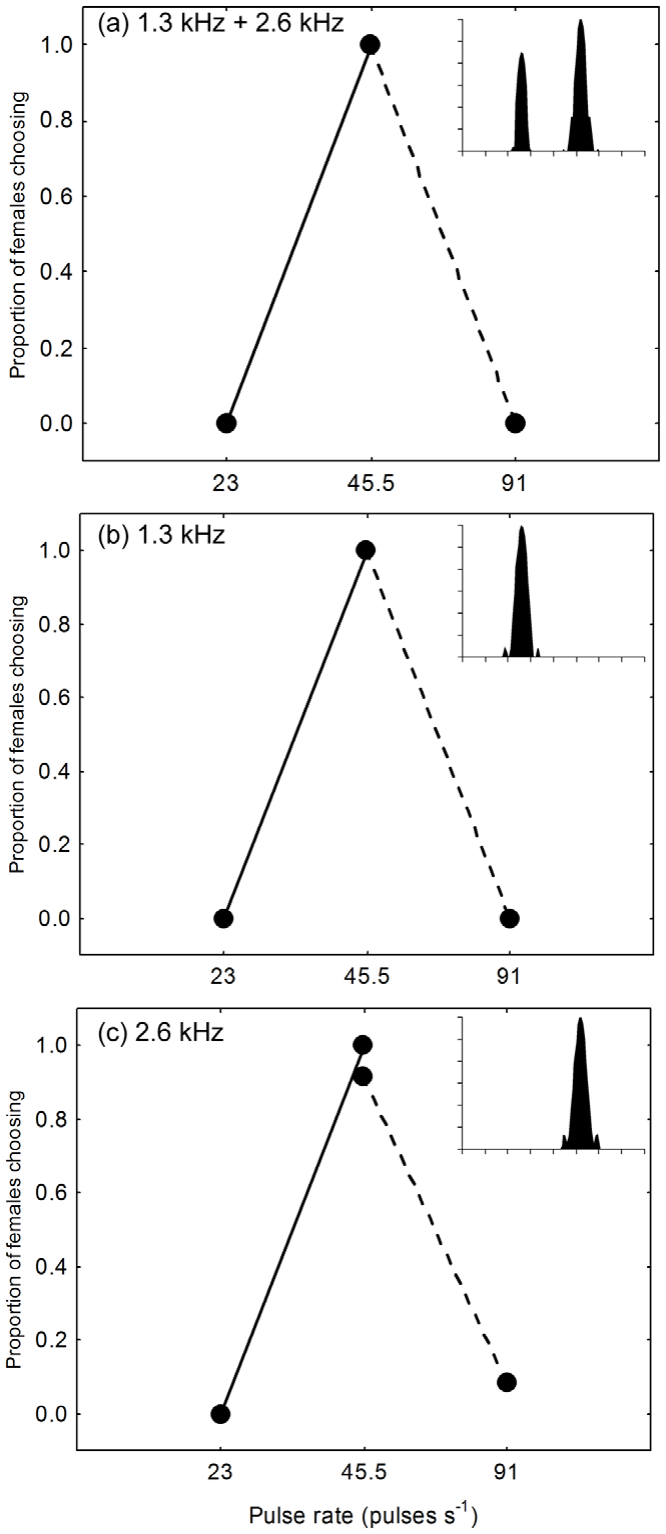
Results of two-choice discrimination tests for pulse rate selectivity. Females were given a choice between two alternating stimuli that differed in pulse rate (see Materials and Methods). Results are shown for tests in which both alternatives had carrier frequencies of (*a*) 1.3 kHz (−9 dB) and 2.6 kHz (0 dB), (*b*) 1.3 kHz, or (*c*) 2.6 kHz. Each line connects two points that show the proportions of females (n = 12 per test) choosing the alternative with a conspecific pulse rate (45.5 pulses s^−1^) and a call with either a slower (23 pulses s^−1^; solid line) or faster (91 pulses s^−1^; dashed line) pulse rate. Insets depict the power spectrum (based on the 45.5 pulses s^−1^ call) for the alternatives in each corresponding two-choice test showing relative amplitude (from 0 dB to −36 dB in 6-dB steps; y-axis) as a function of frequency (from 0 to 4 kHz, 0.5-kHz steps; x-axis). In all tests, females chose the alternative with a conspecific pulse rate significantly more often than expected by chance (two-tailed binomial ps<0.05). These results confirmed that females were selective for conspecific pulse rates with unimodal calls having carrier frequencies of either 1.3 kHz or 2.6 kHz.

**Figure 4:**
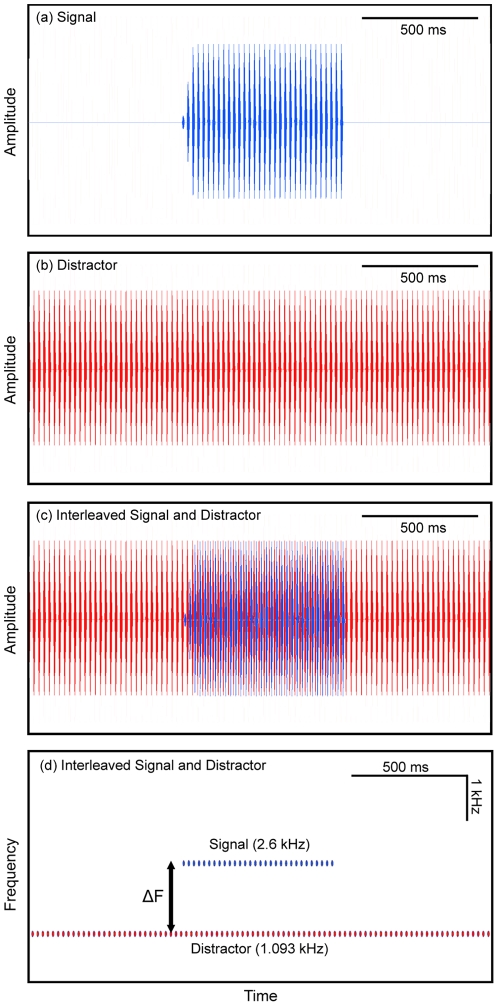
Experimental stimuli for testing the role of ΔF in source segregation. Shown here are examples of (*a*) the waveform of the pulsed target signal; (*b*) the waveform of a 2-s segment of the continuous train of distractor pulses; (*c*) a waveform showing the interleaved target signal and distractor pulses; and (*d*) a spectrogram showing an interleaved target signal (2.6 kHz) and distractor pulses (1.093 kHz) separated by a ΔF of 15 semitones.

**Figure 5:**
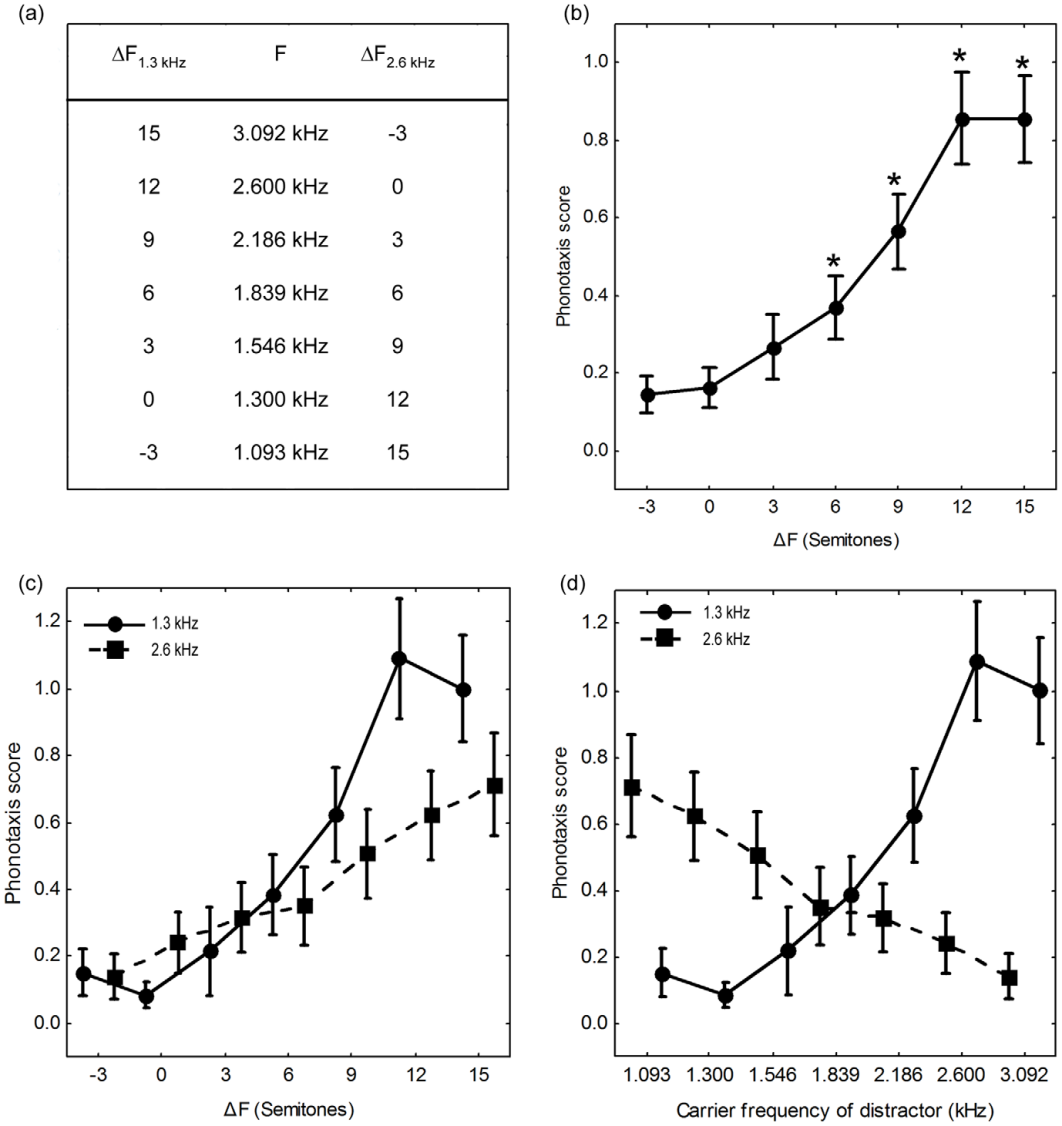
Phonotaxis scores as a function of frequency separation (ΔF) in a test of sound source segregation. (*a*) The absolute carrier frequencies of the distractor pulses (F) shown in relation to the magnitudes of frequency separation (in semitones) for the two target signals with carrier frequencies of 1.3 kHz (ΔF_1.3 kHz_) and 2.6 kHz (ΔF_2.6 kHz_). Note that for the ΔF of 3 semitones, we tested values of absolute frequency that were 3 semitones above and below each signal frequency; we designate these as ΔFs of ±3 semitones, with the positive designation corresponding to the direction of frequency change (either higher or lower) of the other distractor frequencies tested. (*b*) Mean (± SE) phonotaxis scores as a function of ΔF (n = 40). Asterisks indicate significant differences (p<0.05) in planned contrasts comparing the indicated value of ΔF to ΔF = 0. (*c*) Mean (± SE) phonotaxis scores as a function of ΔF shown separately for subjects tested with target signals having a carrier frequency of 1.3 kHz (circles and solid line; n = 20) or 2.6 kHz (squares and dashed line; n = 20). (*d*) Phonotaxis scores from (*c*) plotted as a function of the absolute carrier frequency of the distractor pulses.

In this experiment, we asked whether frequency separation would allow females to recognize an attractive target signal composed of a discrete pulse train simulating the call of a conspecific male presented concurrently with an acoustic “distractor” composed of a continuous pulse train. Our experiment was conceptually similar to previous studies of speech intelligibility in humans in which listeners were asked to recognize short tokens of target speech presented concurrently with longer or continuous speech sounds differing in pitch [8], [9]. We designed our no-choice experiment to exploit (i) the attractiveness of unimodal calls with a single carrier frequency (Fig. 2) and (ii) female preferences for calls with conspecific pulse rates (Fig. 3; [26], [27]). The target signal was a synthetic call with an attractive pulse rate (45.5 pulses s^−1^) and carrier frequency (either 1.3 or 2.6 kHz) (Fig. 4a). This signal was broadcast in the presence of a continuous train of distractor pulses that also occurred at a rate of 45.5 pulses s^−1^ (Fig. 4b), but that was a behaviorally neutral stimulus (see below). The distractor was designed to simulate the pulsatile and often long calls of heterospecific frogs present in mixed-species choruses (Fig. 1) [23]. The target and distractor were presented from the same location, at equal amplitudes, and in such a way that the pulses of the target were temporally interleaved with the pulses of the distractor (Fig. 4c). As a result, the instantaneous pulse rate was 91 pulses s^−1^ at times when the target was presented, but remained a constant 45.5 pulses s^−1^ when only the continuous distractor pulses were broadcast. The carrier frequency of the target was fixed for each subject (either 1.3 kHz or 2.6 kHz). Across trials, we varied the difference (ΔF) between the carrier frequency of the target and that of the distractor over a range of 0 to 15 semitones in 3-semitone steps (Figs. 4d and 5a). The semitone is a common measure of frequency difference used in music and psychophysics and is defined in the equal temperament scale as a frequency ratio of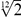; 12 semitones is equivalent to one octave and a 3-semitone difference corresponds to a frequency difference of 18.9%.

Under conditions hypothesized to promote perceptual segregation (e.g., larger ΔFs), we predicted positive phonotaxis because females would perceive the target as a distinct sound source and recognize it as a call with an *attractive* conspecific pulse rate (45.5 pulses s^−1^). If females failed to segregate the target from the distractor (e.g., at smaller ΔFs), they would have experienced an *unattractive* pulse rate (91 pulses s^−1^) each time the target was presented; hence no response would be expected. To test these predictions, we determined “phonotaxis scores” that compared a female's latency to respond to each combination of target and distractor to her latency to respond to an attractive control signal presented by itself during separate reference trials [26], [27], [28]. These phonotaxis scores can be thought of as normalized reaction times in a signal recognition task [28]. A score of 0.0 corresponds to no response occurring within 300 s (5 min); low values correspond to relatively “slow” responses; and values close to 1.0 correspond to typical responses to attractive calls. In no-choice experiments like this one, typical response latencies range between 70 s and 90 s in reference conditions [28], and latencies in this range are generally considered “fast” responses for this species.

Phonotaxis scores were low at small ΔFs and increased as frequency separation increased up to a ΔF of 12 semitones (Fig. 5b). In other words, females were not responsive at small ΔFs and became more responsive (i.e., response latencies became *shorter*) as ΔF increased. In a 7 (ΔF, within-subjects) ×2 (target frequency, between-subjects) ANOVA of phonotaxis scores, we found a large and significant effect of ΔF (F_6,228_ = 17.0, p<0.0001, partial η^2^ = 0.31) and a much smaller but still statistically significant effect due to an interaction between ΔF and the carrier frequency of the target (F_6,228_ = 2.2, p<0.0437, partial η^2^ = 0.05). The main effect of target carrier frequency (1.3 kHz versus 2.6 kHz) was negligible (F_1,38_ = 0.8, p = 0.3823, partial η^2^ = 0.02). Compared to a ΔF of 0 semitones (i.e., the worst case scenario), planned contrasts showed that phonotaxis scores were significantly higher at ΔFs of 6 semitones and larger (6 semitones: F_1,38_ = 5.8, p = 0.0211; 9 semitones: F_1,38_ = 16.1, p = 0.0003; 12 semitones: F_1,38_ = 44.8, p<0.0001; 15 semitones: F_1,38_ = 36.1, p<0.0001), but not at ΔFs of 3 semitones (−3 semitones: F_1,38_ = 0.1, p = 0.7663; +3 semitones: F_1,38_ = 1.3, p = 0.2703). An increase in ΔF beyond 12 semitones had a negligible effect in terms of further increasing female responsiveness (12 vs. 15 semitones: F_1,38_<0.1, p = 0.9984). There was a tendency for phonotaxis scores to increase at a slightly slower rate with increases in ΔF when the signal frequency was 2.6 kHz compared with 1.3 kHz (Fig. 5c). This trend is consistent with the weak interaction between ΔF and target carrier frequency.

The pattern of results shown in Fig. 5b and 5c cannot be explained as a simple function of moving the frequency of the distractor out of the range of best hearing sensitivity. This point is best illustrated in Fig. 5d, which re-plots phonotaxis scores for each signal frequency as a function of the absolute frequency of the distractor pulses (instead of ΔF, as in panel 5c). The key point here is that the pattern of changes in phonotaxis scores as a function of the distractor's absolute carrier frequency *reversed* depending on the carrier frequency of the target signal (Fig. 5d). This reversal indicates that females readily approached either the 1.3 kHz or the 2.6 kHz signal, but only when there was sufficient frequency separation between signal and distractor to segregate one from the other.

### Were the Distractors Behaviorally Neutral?

**Figure 6:**
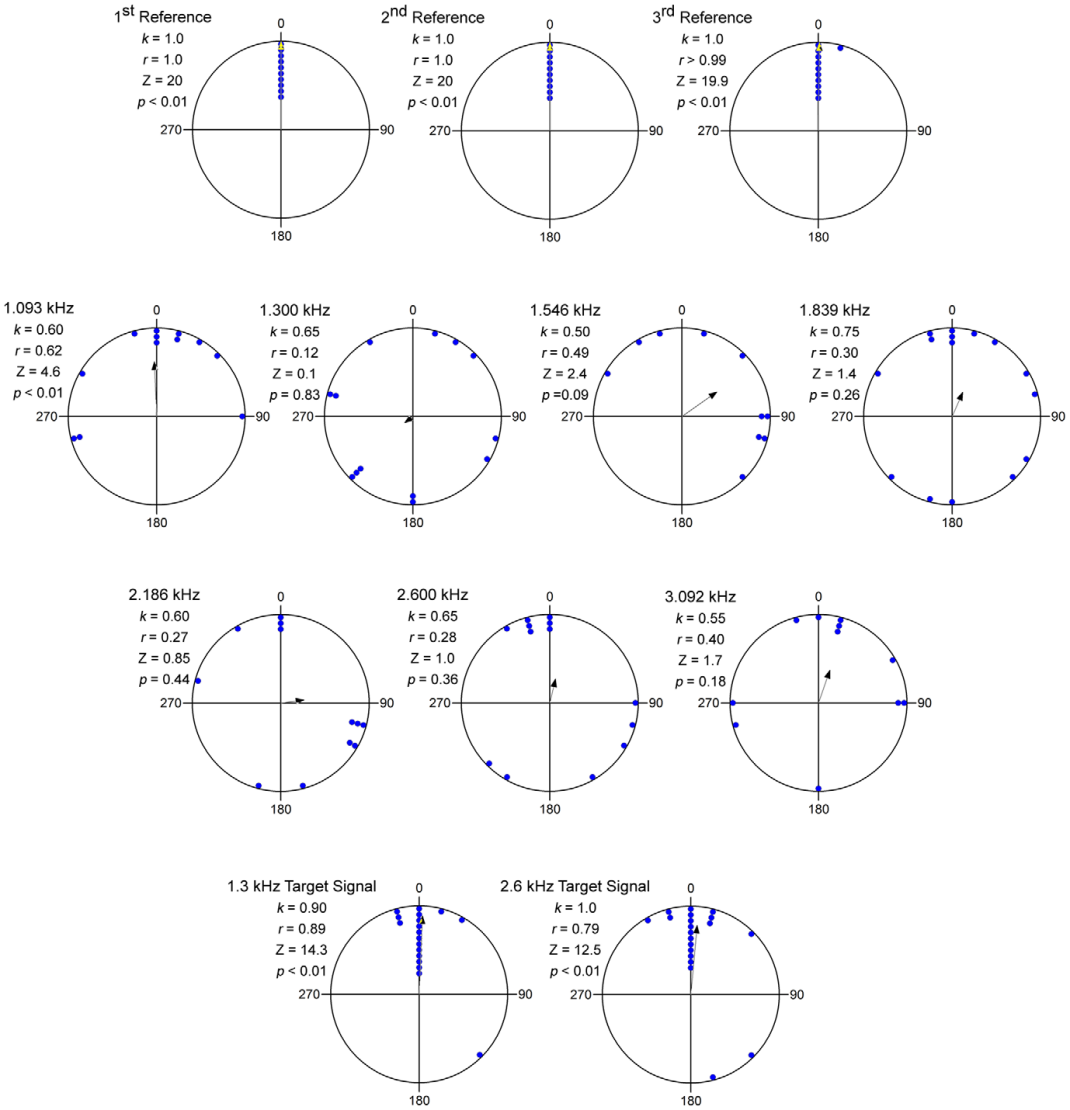
Responses to the distractor stimuli. Each plot shows the distribution of response angles (dots) and the angle and length of the mean vector (arrow) corresponding to the angles at which subjects (maximum possible n = 20 per plot) first touched the wall of the circular test arena relative to the playback speaker positioned at 0°. The text insets show the proportion of subjects that met the response criterion of touching the arena wall during 5 min (k), the length of the mean vector (r), and the results of a Rayleigh test (Z and p) of the null hypothesis that the data are uniformly distributed. Data are shown for the three reference trials tested at the beginning, middle, and end of a sequence of test trials (top row; 1 dot  = 2 subjects), for each of the distractor stimuli (middle rows; 1.093 kHz through 3.092 kHz; 1 dot  = 1 subject), and for subjects tested in the main source segregation experiment in response to the unimodal target stimuli with carrier frequencies of 1.3 kHz or 2.6 kHz presented alone with no distractors (bottom row; 1 dot  = 1 subject).

We conducted a series of no-choice trials to assess female phonotaxis behavior in response to the distractors presented without a target signal. This experiment was conducted to determine whether distractor pulses by themselves had no effect (i.e., a neutral stimulus), or either an attractive or repulsive effect, on female frogs tested in the previous experiment on source segregation. Compared to discrete target signals, responses to continuous trains of distractor pulses were weak and in most cases negligible (Fig. 6). In responses to six of the seven distractor stimuli, there was little indication that subject responses were directed either toward or away from the stimulus. In response to the 1.093 kHz distractor, responses were significantly oriented toward the speaker; however, even in this case, response angles were much more dispersed than responses to presentations of attractive target signals (Fig. 6). In addition, responses were very strongly and significantly oriented toward a speaker broadcasting unimodal targets with carrier frequencies of 1.3 kHz or 2.6 kHz, but not toward distractors with these same carrier frequencies (Fig. 6). Taken together, these results indicate that continuous trains of pulses were generally treated by subjects as neutral stimuli or, at best, very weakly attractive stimuli, compared to discrete pulse trains that mimicked the natural temporal structure of conspecific calls.

## Discussion

Our results indicate female gray treefrogs can segregate concurrent, call-like sounds based on differences in frequency. At smaller ΔFs (e.g., ΔF≤3 semitones), subjects behaved as if they perceptually fused the target signal and distractor pulses into a unified and unattractive percept. As ΔF increased (e.g., ΔF≥6 semitones), however, females increasingly behaved as if they perceptually segregated the signal from the distractor. Because phonotaxis scores represent a continuous measure of signal recognition in frogs [26], our results establish that recognizing conspecific calls in the presence of concurrent call-like sounds improves with increasing frequency separation. Moreover, the significant improvement at 6 semitones has biological significance. The dominant frequency of American toad calls is about 6 semitones higher and lower, respectively, than the 1.3 kHz and 2.6 kHz spectral components present in gray treefrog calls (Fig. 1). This result indicates that frequency separation is one cue that could facilitate the perceptual segregation of conspecific calls from the overlapping calls of other frogs in mixed-species choruses. Interestingly, our results also suggest that frequency separation alone might be an insufficient cue to segregate the overlapping voices of multiple conspecific males, which typically have frequencies within a±3 semitone range. Several important questions remain for future study. For example, what is the influence of ΔF on stream segregation when target signals have a bimodal spectrum, and how does ΔF interact with other potential cues (e.g., differences in pulse rate, amplitude, call duration, or spatial origin)? In natural breeding choruses, we would expect female frogs to exploit variation in multiple different cues to segregate sources of sound. A critical next step in the study of auditory stream segregation in frogs will be to investigate the extent to which ΔF and other potential cues synergistically interact to facilitate signal recognition and biologically relevant discriminations (e.g., between the calls of different species, or those of high-quality and low-quality conspecific males).

Our results are qualitatively similar to those found in previous studies of concurrent speech segregation by humans [8], [9], [10]. For example, using re-synthesized, monotonic speech, Bird and Darwin [9] required listeners to recognize words in a short sentence played during a concurrent longer sentence. Correct word recognition increased from about 20% to above 80% with an increase in ΔF from 0 to 8 semitones. Our results, which are based on using interleaved pulses, are also qualitatively similar to those from other previous studies of source segregation in humans that used simpler, non-speech sounds consisting of interleaved sequences of two short tones differing in frequency (e.g., ABABAB…) [11]. Our auditory system segregates these interleaved tone sequences into separate auditory streams corresponding to separate sequences of pure A or B tones when their acoustic differences are sufficiently large (e.g., ΔF≥6 semitones). Psychophysical studies of goldfish [29], starlings [30], ferrets [31], and monkeys [32] have used similarly simplified stimuli to show that these nonhuman animals also segregate overlapping or interleaved sound sequences into separate auditory streams based on differences in frequency. For example, Izumi [32] showed that monkeys could discriminate between target melodies in the presence of distractor tones only when the distractors were presented in a non-overlapping frequency region. MacDougall-Shackleton et al. [30] found that starlings segregated interleaved triplet tone sequences (e.g., ABA–ABA–…) into separate streams of A and B tones based on frequency separation alone. Fay [29] conditioned goldfish to a mixture of two stimuli, one of which had a high pulse rate and high frequency (625 Hz, 85 pulses per second), while the other had a lower pulse rate and lower frequency (238 Hz, 19 pulses per second). Individuals later tested with high frequency stimuli, generalized to higher pulse rates, while individuals tested with low frequency generalized to lower pulse rates supporting the conclusion that during conditioning, individuals perceived the concurrent stimuli as two different streams. Our results suggest abilities generally comparable to those demonstrated in a few other nonhuman vertebrates are also present in frogs. This study significantly extends these earlier findings by showing that these abilities are potentially exploited by some nonhuman animals to solve real-world communication problems in noisy social environments.

We presently do not know the specific neural mechanisms that allow gray treefrogs to exploit frequency differences in segregating concurrent sounds. Much of auditory scene analysis results from the bottom-up and pre-attentive processing of acoustic cues present in sound mixtures [4]. Electrophysiological recordings in mammalian auditory cortex [33], [34], [35] and its avian homologue [36], [37], [38] have identified frequency selectivity, forward suppression, and neural adaptation as putative physiological correlates of stream segregation [39], and these mechanisms also operate at early stages of the vertebrate auditory pathway [40]. All of these mechanisms have also been described in frogs [41], [42], [43]. Given that frogs lack an auditory cortex and recognize conspecific calls with extensive lesions to thalamic auditory nuclei [44], we suggest the hypothesis that similar low-level neural processes contributed to the source segregation observed in the present study. In particular, we hypothesize that the frequency selectivity of “counting neurons” in the frog midbrain could provide a neural substrate for segregating pulsed mating calls from other sounds. These neurons are frequency selective, exhibit long-term temporal integration, and require presentations of a threshold number of pulses with specific interpulse intervals before firing [45], [46]. The frequency selectivity of counting neurons, combined with their selectivity for the interpulse interval of conspecific calls, could ensure that they only fire when interfering pulses (e.g., those from the distractor) are sufficiently remote in frequency. This hypothesis could be tested in gray treefrogs using target and distractor stimuli similar to those used in the present behavioral study.

An alternative, and non-mutually exclusive, hypothesis to that based on the frequency selectivity of counting neurons involves the possibility that the distractor pulses masked those of the target signal as a result of forward masking (e.g., [41]). According to this hypothesis, as frequency separation increased, the effectiveness of the distractor as a masker should have decreased. Our results cannot exclude this hypothesis. However, to some extent, the operation of auditory masking via forward suppression would be consistent with proposed mechanisms for frequency-based stream segregation in other vertebrates [33], [34], [36], [37]. In addition, the calls of many frogs have pulse rates on the order of 10–100 pulses s^−1^ or higher [23], [47], and recordings from the gray treefrog auditory nerve and midbrain indicate robust encoding of amplitude modulation rates of 50–200 Hz [48], somewhat higher than typically observed in some other vertebrates. Together, these observations suggest that gray treefrogs may have in fact perceived both the target and distractor pulses even when they were interleaved to create a composite pulse rate twice that of conspecific calls.

Examination of the mechanisms that frogs and other nonhuman animals use to segregate overlapping voices is a rich area for future integrative studies of auditory neuroscience, animal communication, and evolution. The ability to segregate overlapping sounds and assign them to different sources based on differences in frequency may be an ancient evolutionary adaptation for hearing that arose in fish and is shared by other vertebrates [49], [50]. In humans, this basic adaptation contributes to our abilities to perceive music and to follow one voice in a multi-talker environment [6]. We suggest that it also contributes to a female frog's ability to selectively attend to the sexual advertisement signals of conspecific males in mixed-species breeding choruses. Additional studies of source segregation in the context of animal communication stand to reveal a great deal about the mechanisms and evolution of sensory systems and their role in generating adaptive behaviors [13].

## Materials and Methods

### Ethics Statement

This study was carried out in strict accordance with the recommendations in the *Guide for the Care and Use of Laboratory Animals of the National Institutes of Health*. Experimental protocols were approved by the University of Minnesota Institutional Animal Care and Use Committee (#0809A46721).

### Subjects

Experiments were conducted between May 15 and July 1, 2008 and 2010, with females of the western mitochondrial DNA lineage [51] collected in amplexus between 2130 and 0200 h from local wetlands (Carver Co., Hennepin Co., and Wright Co., Minnesota, U.S.A.) and returned to the laboratory where they were maintained at 2°C to delay egg deposition until tested. At least 30 minutes prior to the start of a test, subjects were placed in an incubator to allow their body temperatures to reach 20±1°C. After testing, subjects were returned to their location of capture (usually within 48 hrs). A total of 118 females were used as subjects in this study.

### General Procedures

Experiments were conducted in a single-walled, hemi-anechoic sound chamber (L×W×H: 300 cm×280 cm×216 cm; Industrial Acoustics Company) maintained at 20±1°C. Details on the acoustics of the chamber have been described elsewhere [28]. We conducted phonotaxis trials in a circular arena (2-m diameter) with its perimeter divided into 15° bins. The arena wall (60-cm height) was made of hardware cloth covered in black fabric, and was acoustically transparent but visually opaque. We broadcast digital stimuli (20 kHz, 16-bit) from a PC located outside the chamber using Adobe Audition 1.5 interfaced with an M-Audio Firewire 410 soundcard. The soundcard's output was amplified and broadcast using speakers (A/D/S/L210) placed on the chamber floor just outside the arena wall. Speakers were centered in one of the 15° bins and aimed toward a release point in the center of the arena. The frequency response of the playback setup was flat (±3 dB). We calibrated sound pressure levels (SPL re 20 µPa, C-weighted, fast RMS) by placing the microphone of a Larson-Davis System 824 sound level meter at the approximate position of a subject's head at the central release point. All stimuli were created using custom-written scripts in C++ (courtesy J. J. Schwartz) or Matlab v7. In all experiments, the positions of speakers were systematically varied around the circular arena in tests of different subjects to eliminate any possibility of a directional response bias in the data.

To initiate a phonotaxis trial we placed a single subject in a small, acoustically transparent cage at the central release point in the arena. Subjects were initially positioned with random orientation relative to speaker locations and could freely re-orient inside the cage. A trial began with a 1-min silent period for acclimation followed by 45 s of stimulus broadcast while the subject remained in the cage, after which they were remotely released from outside the chamber while the stimuli continued to play. Unless indicated otherwise, subjects were given 5 min to respond by making contact with the arena wall in the 15° bin centered on a speaker broadcasting a signal. All trials were conducted under IR illumination and observed and scored in real time by two observers using a video monitor outside the chamber. Responses were also encoded in real time as digital video files and stored to hard disk. Typically, one observer was blind to the treatment selected by the other observer. Any discrepancies between the two observers in scoring responses were resolved immediately after the trial by watching the recorded video of the trial. In each experiment described below, subjects were tested in 1 to 12 trials with different stimuli and experienced brief “time outs” of 5–15 minutes in the incubator between two consecutive trials. Previous studies of treefrogs have failed to find directional biases or carry-over effects resulting from multiple tests of the same individual [52].

Acoustic stimuli comprised strings of pulses with identical temporal properties approximating average values (corrected to 20°C) from calls recorded in our study populations [28]. A single pulse (11 ms duration) was constructed from either a single sinusoid of constant frequency (for “unimodal” calls), or two phase-locked sinusoids (for “bimodal” calls) at constant frequencies of 1.3 kHz (−9 dB) and 2.6 kHz (0 dB). Unless noted otherwise, target signals comprised 32 consecutive pulses separated by 11-ms inter pulse intervals so that the resulting pulse rate was 45.5 pulses s^−1^ (50% pulse duty cycle; 693 ms signal duration). Target signals were shaped with a 50-ms linear onset and repeated with a period of 5 s, which approximates a natural call rate. In experimental trials involving a distractor, the pulses composing the distractor were broadcast as continuous pulse trains (45.5 pulses s^−1^) over the entire duration of a trial starting after the 1-min acclimation period.

We conducted two types of phonotaxis tests. In “no-choice” tests, we presented subjects with a single target signal. Sometimes target signals in no-choice tests were presented concurrently with distractors. Each series of trials in a no-choice experiment began and ended by testing a “reference trial” in which we presented females with a standard synthetic call of known attractiveness [28] at SPLs of either 79 dB or 85 dB to assess overall response motivation. Reference trials were also re-tested after every third experimental trial in a sequence of experimental trials. We collected data only from subjects that exhibited robust phonotaxis in response to the target signal presented on all reference trials to assure a high level of response motivation across all phonotaxis trials [26], [27], [28]. We also conducted “two-choice” discrimination experiments in which two alternative target signals were alternated in time and broadcast from opposite sides of the test arena. We did not require reference trials to assess female response motivation in two-choice tests because one of the two alternative stimuli was always a signal that elicits phonotaxis from motivated females.

### Audibility Experiment

We presented subjects (total n = 24) with target signals having one of 14 different carrier frequencies. Sounds were broadcast at 67 dB SPL to simulate a male calling at a distance of about 8 m and because this amplitude is well above the threshold sensitivity reflected by midbrain audiograms [24]. One group of 12 subjects was tested with carrier frequencies of 0.5, 1.0, 1.2, 1.4, 2.2, 2.6, and 3.0 kHz in a different randomized order for each subject. A second group of 12 subjects was tested similarly with carrier frequencies of 0.75, 1.1, 1.3, 1.75, 2.4, and 2.8 kHz; eleven of these subjects were also tested with a carrier frequency of 4.0 kHz. We assessed responsiveness using one-tailed binomial tests (α = 0.05) of the null hypothesis that the proportion of subjects responding at each carrier frequency would exceed a previously and empirically determined false alarm rate of 0.2 [28].

### Source Segregation Experiment

The carrier frequency of the target signal used across trials was fixed for a given subject (total n = 40) at either 1.3 kHz (n = 20) or 2.6 kHz (n = 20). Both subject groups were tested with the same set of distractor pulse trains. We manipulated the frequency separation (ΔF) between the signal and distractor by setting the carrier frequency of the distractor used on different trials to 1.093, 1.300, 1.546, 1.839, 2.186, 2.600 or 3.092 kHz (Fig. 5a). These values cover a range of frequencies encompassed in conspecific and heterospecific calls (Fig. 1) and were within the empirically determined hearing range of our study species (Fig. 2). The carrier frequency of the distractor was held constant on a given trial. On one additional trial, the target signal was played back without the distractor. We randomized the order of all trials for each subject. Target signals and distractor pulses were broadcast from two separate but physically adjacent speakers located directly side-by-side. The target signal speaker was centered in the 15° bin of the test arena. The position of the speaker broadcasting distractor pulses (left or right relative to the target) was varied randomly across tests of different subjects. The signal and all distractors were separately calibrated to be 67 dB SPL as in the audibility experiment.

We scored a response when three conditions were met: (i) the subject's first contact with the arena wall was in the same hemi-circle as the target speaker, (ii) the subject touched the arena wall in the 15° bin centered on the target speaker within 5 min of being released, and (iii) after touching the wall at this bin she remained for 30 consecutive seconds within 20 cm of the arena wall inside a bin of 30° centered on the target speaker. To measure signal recognition as a continuous variable, we determined phonotaxis scores [26], [28] by normalizing a subject's latency to respond to each signal+distractor combination relative to its average response latency on the two most temporally adjacent reference conditions in a test series. Subjects that failed to meet all three response criteria were assigned a score of zero. We obtained qualitatively similar results in separate statistical analyses in which phonotaxis scores were computed by normalizing response latencies to a subject's latency to respond to the target signal presented alone at 67 dB.

### Distractor Neutrality Experiment

Across a sequence of seven different test trials, we presented females (n = 20) with each of the distractor stimuli in different randomized orders. All distractor stimuli were calibrated to be 67 dB SPL and had carrier frequencies of 1.093, 1.300, 1.546, 1.839, 2.186, 2.600, or 3.092 kHz. Trials were terminated when the females reached the arena wall and we noted the angle at which subjects first touched the wall relative to the speaker, which had a designated position of 0°. We also tested reference trials using a standard, attractive call (85 dB SPL) [28] as the first and last trials of a sequence, and in the middle of the sequence after the third or fourth test trial with the distractor stimuli. We used circular statistics (Rayleigh tests) to test the null hypothesis that response angles were uniformly distributed against the alternative hypothesis that the orientations of responses were grouped in space. We used non-directional Rayleigh tests, instead of V tests of the directional hypothesis that responses would be oriented toward the sound source at 0°, in consideration of the possibility that females also could have orientated *away* from the sound source.

### Pulse Rate Selectivity Experiment

Previous studies have shown female gray treefrogs to be selective for conspecific pulse rates using “bimodal” calls with both of the two dominant spectral components present [26], [27]. Our test of sound source segregation was designed to exploit this pulse rate selectivity using “unimodal calls” with a single carrier frequency. We, therefore, conducted two-choice discrimination experiments to confirm that females from our study populations were selective for conspecific pulse rates in response to hearing both unimodal and bimodal calls. We gave subjects a choice of two stimuli that differed in pulse rate (constant pulse durations and shapes; variable inter-pulse interval and pulse number). The two stimuli alternated in time between two speakers located 2 m and 180° apart around the circular test arena. In separate tests, we paired a signal with a conspecific pulse rate (45.5 pulses s^−1^; 32 pulses) against an alternative with either a slower (23 pulses s^−1^; 16 pulses) or faster (91 pulses s^−1^; 64 pulses) pulse rate. Each alternative repeated with a period of 5 s, and the two alternatives were alternated so that each was preceded and followed by equal periods of silence. Both two-choice tests were replicated using three different types of paired signals differing in spectral content. In one replicate, we alternated two bimodal calls. In the two remaining replicates, we alternated two unimodal calls with carrier frequencies that were either both 1.3 kHz or both 2.6 kHz. All stimuli were presented at 67 dB SPL. We scored a subject's choice when it first made contact with the arena wall in the 15° bin centered in front of one of the playback speakers. We used two-tailed binomial tests to compare the observed proportions of females (n = 12 per test) choosing the alternative with the conspecific pulse rate to the null expected proportion of 0.50 (α = 0.05).
